# Associations of physical activity and fruit and vegetable intake with well-being and depressive symptoms among obese schoolchildren in Wuhan, China: a cross-sectional study

**DOI:** 10.1186/s12889-018-5779-9

**Published:** 2018-08-08

**Authors:** Hong-jie Yu, Fang Li, Yong-feng Hu, Chang-feng Li, Xu-hao Yang, Shuai Yuan, Yao Huang, Bo-wen Tang, Jie Gong, Qi-qiang He

**Affiliations:** 1 School of Health Sciences, Wuhan University, Donghu Road 185#, Wuhan, People’s Republic of China; 2 Wuhan Centers for Disease Prevention and Control, Hanbei Road 24#, Wuhan, People’s Republic of China; 3 Xinzhou Centers for Disease Prevention and Control, Wuhan, People’s Republic of China

**Keywords:** Physical activity, Fruit and vegetable intake, Obesity, Children, Mental health

## Abstract

**Background:**

The prevalence of childhood obesity is increasing and psychological disorder is a common comorbidity of obesity. We investigated the associations of physical activity (PA) and fruit and vegetable (FV) intake with well-being and depressive symptoms among obese schoolchildren.

**Methods:**

Participants included 188 obese children aged 9.8 ± 0.7 years living in Wuhan, China. Self-administered questionnaires were used to collect the children’s PA and FV intake information. PA was considered to be high if the child participated in sport and/or vigorous free play at least 3 days per week with 60 min per day, while sufficient FV intake was defined as consuming FV 5 times per day. Children’s well-being and depressive symptoms were assessed by standard questionnaires. Multiple logistic regression was performed to determine the odds ratios (ORs) and 95% confidence intervals (CIs) of the relationships of PA and FV intake with well-being and depressive symptoms.

**Results:**

High PA and sufficient FV intake were independently associated with significantly decreased risks for depressive symptoms (for PA, OR: 0.39, 95% CI: 0.16–0.92; for FV, OR: 0.21, 95% CI: 0.08–0.55) and poor well-being (for PA, OR: 0.35, 95% CI: 0.16–0.74), respectively. Furthermore, interactive inverse associations were observed between combined high PA and sufficient FV intake with poor well-being and depressive symptoms. Compared to their counterparts, children with high PA and sufficient FV intake had significantly reduced risk for poor well-being (OR: 0.16, 95%CI: 0.05–0.55) and depressive symptoms (OR: 0.12, 95% CI: 0.03–0.48).

**Conclusions:**

High PA and sufficient FV intake are inversely associated with the risks of poor well-being and depressive symptoms among obese Chinese schoolchildren.

**Electronic supplementary material:**

The online version of this article (10.1186/s12889-018-5779-9) contains supplementary material, which is available to authorized users.

## Background

The increasing prevalence of childhood obesity has become a serious public health problem worldwide [1, 2]. A recent report by World Health Organization indicated that the global prevalence of overweight and obesity increased dramatically from 4% in 1975 to over 18% in 2016 among children and adolescents aged 5–19 years [3]. Likewise, results from a nationally representative sample of 40,780 US children and adolescents suggested that the prevalence of obesity among children aged 6 to 11 years increased from 11.3% in 1988–1994 to 17.4% in 2012–2013 [4]. In China, rapid economic growth has resulted in an increased prevalence of obesity during the last few decades. Findings from a national survey between 1997 and 2011 showed that the prevalence of obesity in children aged 7–17 years more than doubled from 6.5 to 15.5% in boys and from 4.6 to 10.4% in girls [5].

Childhood obesity has been linked to increased metabolic and cardiovascular risks [6]. In addition, cumulative evidences indicate that mental disorder is a common comorbidity of obesity [7, 8]. The prevalence of depressive symptoms in children and adolescents was approximate to 14% in China [9], and that in obese children and adolescents even reached to 23.62% [10]. Findings from a 2-year longitudinal study also suggested a significantly increased risk for poor well-being among overweight and obese children [11]. Given that obesity and obesity-related psychological comorbidities may continue from childhood into adulthood [6, 12], it is of importance to initiate early interventions to prevent obesity and reduce the risk of mental disorders in young children.

Unhealthy lifestyles including low physical activity (PA) and insufficient fruit/vegetable (FV) intake are very common among children and adolescents, and play a crucial role in the development of obesity [13]. Few studies have examined the association of PA and FV intake with mental health in general child population. The results from the Canadian Community Health Survey indicated that sufficient FV intake was associated with a 27% decrease in odds of depressive symptoms relative to control group [14]. A meta-analysis pooling 73 studies also demonstrated significant effects of PA on children’s mental health [15]. However, it is unclear whether PA and FV intake are positively associated with mental health among Chinese children with obesity, although the relationships of these factors with physical health have been well documented [16, 17]. This information could be useful to develop interventions to inhibit the increasing prevalence of childhood obesity in China [18]. Therefore, the main objective of this study was to examine the associations of PA and FV intake with mental health in a sample of obese Chinese schoolchildren.

## Methods

### Study design and participants

This cross-sectional study was conducted in November 2015 in Wuhan, China. One district (Xinzhou district) was randomly selected from 13 districts in Wuhan city. Then two schools from 30 public primary schools in Xinzhou District were randomly recruited for this study. Sample size was calculated by the following equation: *n* = *z*^2^*p(*1*-p*)/*d*^2^, where *n* = sample size, *z* = *z* statistic corresponding to a chosen level of confidence (1.96), *p* = expected prevalence (0.13), and *d* = precision (0.05) [19], considering non-response rate of 5%, this calculation resulted in a sample size of 183 obese children. Children in Grade 3rd and 4th in each school were invited to participate in a physical examination (height and weight measures). Of 1340 children (1051 boys), 203 obese children (152 boys) were identified using Chinese body mass index (BMI) cut-off points (Additional file 1: Table S1) [20]. Written informed consent was obtained from parents of the children. Only children who consented and obtained their parents’ consent were included in this study. Children with a known chronic disease (such as diabetes, heart diseases, etc.) were excluded. This study was approved by the Medical Research Ethics Committee of Wuhan University.

### Measures

Children completed a self-administered questionnaire in classrooms with the guidance of investigators who were trained medical graduate students from Wuhan University. The questionnaire contained information on their PA, FV intake, poor well-being and depression status. Parents were asked to report their household income using a short questionnaire.

#### Physical activity

Habitual PA was asked by the question - How often do you sport and/or vigorous free play (such as running, gymnastics, push-ups and jumping rope) each week with 60 min at least per day? Response options included: less than once per week, 1–2 days/week, 3–4 days/week, 5–6 days/week, and daily. This item was derived from the PA recommendation for children published by US Centers for Disease Control and Prevention [21], which was a valid instrument for examination of PA and basically consistent with PA guidelines for Chinese Children published by Working Group on Physical Activities Guidelines for Children and Adolescents in China [22]. Children with high PA were defined as those who participated in sports and/or vigorous free play at least three days per week for at least 60 min per day.

#### Fruit and vegetable intake

FV intake was assessed by the question - During the past 30 days, how many times per day did you usually eat fruits/vegetables? There were seven possible options: I did not eat fruit/vegetables, less than once per day, once per day, 2 times per day, 3 times per day, 4 times per day, 5 or more times per day. The FV questionnaire was derived from Global School-based Student Health Survey, and its reliability and relative validity has been assessed among school-aged children in China. Children with sufficient FV intake were defined as those who consumed at least 5 times per day [23].

#### Well-being

Well-being was assessed by the WHO-5 well-being index (WHO-5), which is the most widely used questionnaire measuring subjective well-being, and its good reliability and validity have been shown in children [24]. The questionnaire consists of 5 positively worded items that reflect the presence or absence of well-being in the past 2 weeks: (1) I have felt cheerful and in good spirits, (2) I have felt calm and relaxed, (3) I have felt active and vigorous, (4) I woke up feeling fresh and rested, and (5) My daily life has been filled with things that interest me. Each item score ranges from 0 to 5, and the sum scores is between 0 and 25. A total score < 13 indicates poor well-being [24].

#### Depressive symptoms

Depressive symptom was evaluated using the Depression Self-rating Scale for Children (DSRSC). DSRSC has been widely used to assess depressive symptoms among children, and its reliability and validity have been examined in Chinese children [25]. It consists of 18 items utilizing a three-point scale response format including “never (score = 0)”, “sometimes (score = 1)” “most of the time (score = 2)”, and the sum score is between 0 and 36. Higher scores on the DSRSC indicate a higher level of depression, and a total score of 15 is set as a cut-off point of depressive disorders to divide participants into two groups (yes/no) [25].

### Statistical analysis

Study children were divided into low (insufficient) and high (sufficient) groups in accordance with the PA level and FV intake, respectively. There was an interactive effect between PA, FV intake and the risk of psychological factors in the study children (*p* <  0.05). Each socioeconomic variable and BMI were initially checked separately with main model by psychological status, only statistical significant variables were entered in the final model as potential confounders (school, sex, age, BMI, and household income). Multiple logistic regression models were performed to examine the odds ratios (ORs) and 95% confidence intervals (CIs) of the independent and interactive relationships of PA and FV intake with poor well-being and depressive symptoms. Hosmer and Lemeshow test were applied to assess the goodness of fit and Nagelkerke R^2^ values was used to estimate the proportion of explained variance in the logistic regression models. Statistical analyses were conducted using the SPSS statistical package (version 13.0; SPSS Inc., Chicago, IL. USA).

## Results

A total of 188 obese children was included in the present study, including 42 (22.3%) girls and 146 (77.7%) boys. Table 1 displays the basic characteristics of participants with an age of 9.8 years (standard deviation, SD = 0.7). Children who reported high PA and sufficient FV intake accounted for 36.7 and 45.7%, respectively. Forty three (22.9%) children were identified with poor well-being, and 46 (24.5%) children had depressive symptoms.

**Table 1:** Basic characteristics of participants

Characteristics	
Age (years), mean, SD	9.8(0.7)
Sex-girls, %	22.3
Height (cm), mean, SD	144.8(5.9)
Weight (kg), mean, SD	51.0(7.1)
BMI (kg/m^2^), mean, SD	24.3(2.2)
Monthly household income, %	
Low(< 5000 Yuan RMB)	38.8
Middle(5001–10,000 Yuan RMB)	37.8
High(> 10,000 Yuan RMB)	14.4
Physical activity, %	
High	36.7
Low	63.3
Fruit and vegetable intake, %	
Sufficient	45.7
Insufficient	54.3
Poor well-being, %	22.9
Depressive symptoms, %	24.5

*BMI* body mass index, *Yuan RMB* unit of Chinese money, *SD* standard deviation

Data are presented as mean (SD) or percentage, where appropriate

The independent associations of PA and FV intake with well-being and depressive symptoms are shown in Table 2. After adjustment for several potential confounders, high PA was associated with significantly reduced risks for depressive symptoms (OR: 0.39, 95% CI: 0.16–0.92,*p* <  0.05) and poor well-being (OR: 0.35, 95% CI: 0.16–0.74, *p* < 0.01), respectively. Sufficient FV intake was associated with 79% reduction (OR: 0.21, 95% CI: 0.08–0.55, *p* < 0.01) in the risk of depressive symptoms, while the association between FV intake and poor well-being was statistically insignificant.

**Table 2:** Associations between physical activity, fruit/vegetable intake and well-being, depressive symptoms among Participants

Characteristics	Depressive symptoms		*p*-value	Poor well-being		*p*-value
	N (%)	OR (95% CI)		N (%)	OR (95% CI)	
Physical activity^a^						
Low	33 (17.5)	1.00		36 (19.1)	1.00	
High	10 (5.32)	0.39 (0.16–0.92)	0.031	10 (5.3)	0.35 (0.16–0.74)	0.006
Fruit and vegetable intake^b^						
Insufficient	26 (13.8)	1.00		24 (12.8)	1.00	
Sufficient	17 (9.0)	0.21 (0.08–0.55)	< 0.001	22 (11.7)	0.57 (0.28–1.18)	0.130

Abbreviation: *OR* odd ratio, *CI* confidence interval

Adjusted for school, sex, age, BMI, and household income

^a^For depressive symptoms: Hosmer and Lemeshow Test: Chi^2^ = 6.42, *p* = 0.601, Nagelkerke R^2^ = 0.284

^b^For depressive symptoms: Hosmer and Lemeshow Test: Chi^2^ = 7.74, *p* = 0.460, Nagelkerke R^2^ = 0.273

^a^For poor well-being: Hosmer and Lemeshow Test: Chi^2^ = 5.45, *p* = 0.709, Nagelkerke R^2^ = 0.403

^b^For poor well-being: Hosmer and Lemeshow Test: Chi^2^ = 4.88, *p* = 0.770, Nagelkerke R^2^ = 0.382

Table 3 shows the interactive effects of PA and FV intake with well-being and depressive symptoms. Compared with those who had low PA and insufficient FV intake, children with high PA/insufficient FV had 79 and 73% lower risk of poor well-being and depressive symptoms (all *p* < 0.05), respectively, while children with high PA/sufficient FV had the lowest risk for poor well-being (OR: 0.16, 95% CI: 0.05–0.55, *p* < 0.01) and depressive symptoms (OR: 0.12, 95% CI: 0.03–0.48, *p* < 0.01). The total explained variance for depressive symptoms and poor well-being, according to Nagelkerke R^2^, was 29.0 and 40.4%, respectively.

**Table 3:** Multiple logistic regression for well-being and depressive symptoms by combined physical activity and fruit/vegetable intake

Mental health	Fruit and vegetable intake	Low physical activity		*p*-value	High physical activity		*p*-value
		N (%)	OR (95% CI)		N (%)	OR (95% CI)	
Depressive symptoms^a^	Insufficient	21 (11.2)	1.00		5 (2.7)	0.21 (0.06–0.77)	0.019
	Sufficient	12 (6.4)	0.57 (0.24–1.32)	0.185	5 (2.7)	0.12 (0.03–0.48)	0.003
Poor well-being^b^	Insufficient	19 (10.1)	1.00		5 (2.7)	0.27 (0.08–0.88)	0.028
	Sufficient	17 (9.0)	0.27 (0.11–0.68)	0.005	5 (2.7)	0.16 (0.05–0.55)	0.003

*OR* odd ratio, *CI* confidence interval

Adjusted for school, sex, age, BMI, and household income

^a^For depressive symptoms: Hosmer and Lemeshow Test: Chi^2^ = 5.98, *p* = 0.650, Nagelkerke R^2^ = 0.290

^b^For poor well-being: Hosmer and Lemeshow Test: Chi^2^ = 7.10, *p* = 0.526, Nagelkerke R^2^ = 0.404

## Discussion

Results from this study indicated that PA and FV intake were independently associated with reduced risk of poor well-being and depression among obese Chinese schoolchildren. Furthermore, obese children with high PA and sufficient FV intake had over 80% lower odds of poor well-being and depressive symptoms compared with those with low PA and low FV intake.

Previous studies revealed a relatively higher prevalence of psychological problems among obese children compared with that of this study. For example, in a sample of 102 adolescent patients from a weight management clinic, 34% had depressive symptoms and 32% had symptoms of anxiety [26]. A cross-sectional study in Canada found that depressive symptoms were common (36.4%) in youth with obesity [27]. A systematic review of nine studies summarized that childhood obesity was related to several mental health disorders, including depression, low self-esteem and poor health-related quality of life among Australian children [28]. Two prospective cohort studies also found that adolescent obesity or overweight predicted subsequent depression and/or anxiety in adulthood [29, 30]. Therefore, in the design of effective approaches for preventing and treating obesity, it is of importance to consider whether adequate PA and sufficient FV intake can also improve psychological problems in obese children.

Previous investigations revealed that low PA and insufficient FV intake were very common among obese children. In the present study, a high percentage of children did not meet the recommendations for PA (63.3%) and FV intake (54.3%). Furthermore, we found that low PA and insufficient FV intake had a strong relationship with poor well-being and depressive symptoms. Although some studies reported no significant association between PA/FV and mental health among children and adolescents [31, 32], our results were generally in accordance with most previous epidemiological studies [33, 34]. For example, a follow-up study found that PA might be more beneficial for mental functioning among those with overweight than normal weight individuals [33]. Evidence from a Canadian national survey also suggested that the consumption of FV played an important role in the prevention of mental disorders [35]. These findings suggest that high PA and sufficient FV intake recommendation should be integrated into mental health prevention strategies to reduce the risk of psychological problems among children.

Although healthy lifestyle behaviors have been associated with better cardiovascular and metabolic health [36], their joint associations with mental health have been little studied. In a recent cohort study, combined effects of PA and healthy dietary patterns on metabolic syndrome were observed among Chinese adults [37]. Nevertheless, the joint relationship of PA and FV intake with obese children’s mental health remained unclear. The present study not only shows an independent favorable influence of PA and FV intake, but also reveals a combined association of high PA and FV intake with reduced risk of poor well-being and depressive symptoms among obese Chinese children. Thus, our results add further evidence for future prevention and health promotion strategies integrating PA and FV consumption to promote mental health among obese children.

The exact mechanisms of synthetic effects of PA and FV on mental health have yet to be fully elucidated. Exercise can stimulate the growth of new nerve cells and the induction of the release of proteins and peptides that promote the sense of well-being [38]. Meanwhile, FV are abundant in micronutrients and phytochemicals which may not only mediate anti-oxidative and anti-inflammatory activities [39], but also improve the release of serotonin in the brain [14]. In addition, dietary factors may exert their effects on brain through influencing molecular events associated with cell and energy metabolism, which affects neuronal function and signaling, thus modulating mental health [40]. In terms of behavioral level, the latest research performed by Lena et al. reported that physical activity and nutrition appeared to facilitate rather than hindered each other among participants, and there was a “facilitating pattern” between PA and FV intake [41]. Since both PA and FV intake have been proven to improve psychological well-being through several pathways, it is biologically plausible that the combination of high PA and sufficient FV intake may contribute a better mental health.

The present study has several limitations. First, given that the cross-sectional study design cannot infer the causality of PA and FV intake with poor well-being and depressive symptoms, PA and FV intake may be a result of the nutritional and psychological status of the children and not the opposite. Second, international standardized questionnaires have been used to assess mental health. However, these measures were not equivalent to clinical diagnoses. Third, the FV questionnaire is a crude measure of food consumption. The items focused on frequency rather than portion size, and the questionnaire has not been validated in children. Nevertheless, it is simple and convenient to complete, and has been previously used among Chinese children [42]. Fourth, PA levels were self-reported by children, and thus recall and reporting bias might exist. Furthermore, we did not specify vigorous-intensity PA and moderate-intensity PA in the questionnaire. However, the aim of this study was to compare high PA with low PA, rather than PA intensity, on the risk of mental health. In addition, self-report PA has been widely used in epidemiological studies [43, 44]. The question for PA was similar to several previous studies that we conducted among Chinese schoolchildren [45, 46]. In addition, PA recalls are reliable estimate of PA in research among young children [47]. Fifth, although the reliability and validity of WHO-5 well-being index have been examined in western pediatric population, there is a lack of similar research in Chinese children. Thus, potential bias cannot be excluded. Last, only two schools from one district were included in this study, and there was a wide difference in the numbers of male and female participants. Therefore, caution should be made when generalizing our findings to national or international levels given the exclusive geographic location of this study.

## Conclusions

In summary, results from the present study suggested that high PA and sufficient FV intake were inversely associated with poor well-being and depressive symptoms among Chinese obese children and showed additive associations. Our study provides further support for the key role of PA and FV intake in the prevention of mental disorders among obese children.

## Additional file

Additional file 1: **Table S1.** Cut-off points of body mass index for obese children aged 8–12 years in China. (DOC 30 kb)

## Abbreviations

BMI: Body mass index
CI: Confidence interval
DSRSC: Depression self-rating scale for children
FV: Fruit and vegetable
OR: Odds ratio
PA: Physical activity
SD: Standard deviation
