# Supplementary material for: Associations of physical activity and fruit and vegetable intake with well-being and depressive symptoms among obese schoolchildren in Wuhan, China: a cross-sectional study
Source: BMC Public Health. 2018 Aug 8;18:986. doi: 10.1186/s12889-018-5779-9 (PMC6083616; doi:10.1186/s12889-018-5779-9)
Supplement: Supplementary file 1 — Table S1. Cut-off points of body mass index for obese children aged 8–12 years in China. (DOC 30 kb) [file 12889_2018_5779_MOESM1_ESM.doc]

**Additional file 1: Table S1** Cut-off points of body mass index for obese children aged 8-12 years in China^*^

| Age (year) | Boys (kg/m^2^) | Girls (kg/m^2^) |
| --- | --- | --- |
| 8 | 20.3 | 19.9 |
| 9 | 21.4 | 21.0 |
| 10 | 22.5 | 22.1 |
| 11 | 23.6 | 23.3 |
| 12 | 24.7 | 24.5 |

* Ji CY, Working Group on Obesity in China: Report on childhood obesity in China (1)--body mass index reference for screening overweight and obesity in Chinese school-age children. Biomed Environ Sci 2005, 18(6):390-400.
